# Genomic characterization and pathogenicity assessment of a novel strain of duck reovirus identified in China

**DOI:** 10.3389/fmicb.2026.1750966

**Published:** 2026-03-17

**Authors:** Xiuling Wang, Kun Yan, Mengjiao Guo, Zongyi Bo, Xiaorong Zhang, Chengcheng Zhang, Yantao Wu

**Affiliations:** 1 Jiangsu Co-Innovation Center for the Prevention and Control of Animal Infectious Disease and Zoonoses, College of Veterinary Medicine, Yangzhou University, Yangzhou, Jiangsu, China; 2 International Joint Research Laboratory of Agricultural and Agri-Product Safety, The Ministry of Education of China, Yangzhou University, Yangzhou, Jiangsu, China

**Keywords:** genome sequence, global distribution, novel duck reovirus, pathogenicity, phylogenetic analysis

## Abstract

The global duck industry faces substantial challenges due to the emergence of novel duck reovirus (NDRV) infections, which are pathologically characterized by splenomegaly, hemorrhagic manifestations, and necrotic lesions in affected birds. Surviving ducklings often suffer from severe growth retardation. Here, we investigated an outbreak of hepatic necrosis in commercial ducklings from Shandong Province, China. After excluding other potential pathogens, an NDRV strain was isolated and designated SD416. This strain demonstrated infectivity in both chicken and duck embryos and induced syncytial formation in Vero cells. Full-genome sequencing revealed a 23,420-bp dsRNA genome consisting of 10 segments, displaying significant genetic divergence from other Chinese duck reovirus isolates. Phylogenetic analysis indicated that SD416 is genetically distinct from known DRV strains, particularly within the M2 and S3 genes. Furthermore, its σC protein-coding sequence exhibited notably high genetic variability compared to strains from other genotyping clusters. To evaluate the age-dependent pathogenicity of SD416, 1-, 7-, and 14-day-old ducklings were intramuscularly inoculated with 0.1 mL of allantoic fluid containing the virus at a titer of 1 × 10⁶ TCID₅₀/0.1 mL. The strain exhibited pronounced age-related virulence: 1-day-old ducklings developed severe hepatic and splenic lesions resulting in mortality, whereas ducklings inoculated at 7 and 14 days of age exhibited only splenic pathology and survived throughout the study. The SD416 strain was comprehensively characterized in terms of tissue tropism, pathogenesis, genomic structure, and evolutionary relationships. Our findings reveal a unique genomic and virulence pattern associated with this novel isolate, providing important insights into the biology of NDRV. These results underscore the need for enhanced surveillance and development of targeted intervention strategies to limit the spread of this virus in global waterfowl production systems.

## Introduction

Novel duck reovirus (NDRV), a member of the genus *Orthoreovirus* within the family *Reoviridae*, causes severe hemorrhagic necrotic lesions in the liver and spleen of various duck species, thereby representing a substantial threat to the global waterfowl industry (Attoui et al., 2000; Yun et al., 2014; Zhang et al., 2021; Zhong et al., 2016). The disease was first described in Muscovy ducks (*Cairina moschata*) in 1950 (Kaschula, 1950). The causative agent, later isolated and designated classical Muscovy duck reovirus (MDRV), primarily infects 10-day-old Muscovy ducklings, with susceptibility lasting up to 6 weeks of age. Infection is characterized by hepatic and splenic enlargement, hemorrhage, and multifocal necrotic lesions. Since 2005, a distinct reovirus associated with increased mortality and marked splenic necrosis has been identified in deceased Pekin ducklings (*Anas platyrhynchos domestica*) (Liu et al., 2011). This pathogen, which exhibits pathogenic properties distinct from classical MDRV, was designated the novel duck reovirus (NDRV) (Doyle et al., 2015; Heggen-Peay et al., 2002). NDRV demonstrates a broader host range, capable of infecting multiple duck and goose species. Susceptibility and mortality rates are inversely correlated with host age, with younger birds exhibiting significantly higher fatality rates. Furthermore, the incidence of NDRV infection has increased annually in recent years (Li et al., 2018; Zhu et al., 2015).

NDRV is a non-enveloped virus with an icosahedral capsid and a double-stranded RNA genome. The genomic RNA consists of 10 segments, which can be categorized by size into three classes based on electrophoretic mobility: large (L1–L3), medium (M1–M3), and small (S1–S4) (Farkas et al., 2021; Niu et al., 2018a; Zhang et al., 2019). These segments collectively encode at least 12 viral proteins, including structural components such as σC, σB, σA, and σNS, which play critical roles in viral attachment, cell entry, and immune evasion (Noad et al., 2006; Varela and Benavente, 1994). Genomic plasticity, particularly in genes encoding outer capsid proteins, contributes to the significant genetic diversity and evolving antigenic characteristics of NDRV. The σC protein, a major determinant of viral neutralization and cell tropism, displays considerable sequence variation among strains. Recent phylogenetic analyses indicate that NDRV isolates form distinct genetic lineages, with evidence of ongoing evolution and potential recombination events with other avian *orthoreoviruses*, such as Muscovy duck reovirus (MDRV). This genetic variability challenges the development of universal diagnostic assays and effective vaccines (Yang et al., 2023; Zheng et al., 2016).

Current surveillance data indicate that NDRV has an expanding global distribution, with the highest prevalence in East and Southeast Asia, particularly in China (e.g., Shandong, Hebei, and Henan provinces). Sporadic reports also suggest its potential presence in Europe and North America, likely linked to international poultry trade (Kong et al., 2023; Varga-Kugler et al., 2022). Due to its high morbidity and mortality rates and broad host specificity, NDRV has become one of the most significant infectious diseases affecting duck production in China (Yan et al., 2021). Therefore, sustained surveillance is essential for monitoring circulating and emerging NDRV strains in waterfowl populations, a critical step in developing targeted prevention and control strategies (Wang S. et al., 2020).

In this study, a novel duck *orthoreovirus* strain, designated SD416, was isolated from the liver of diseased ducklings from a commercial farm in Shandong Province, China. We performed epidemiological tracing, pathogen isolation and identification, and whole-genome sequencing. In addition, both *in vitro* and *in vivo* assays were conducted to evaluate the pathogenicity and underlying mechanisms of the isolate in young ducks. This study aims to enhance our understanding of the pathogenic mechanisms of strain SD416 and provide new insights into the evolutionary relationships of potentially recombinant viruses across host species.

## Materials and methods

### Animals and ethics statement

Specific pathogen-free (SPF) ducks were supplied by Shandong Haotai Experimental Animal Breeding Co., Ltd. and housed in negative-pressure isolators equipped with high-efficiency particulate air (HEPA) filtration. All animal experiments were approved by the Animal Welfare and Ethical Review Committee of Yangzhou University (Approval No. 202503268, dated March 26, 2025) and conducted in accordance with the guidelines set forth in the Guide for the Care and Use of Agricultural Animals in Research and Teaching (4th edition, 2020).

### Virus isolation and identification

Five liver tissue samples displaying characteristic pathological lesions were obtained from a commercial Cherry Valley duck farm in Shandong Province in April 2023. The affected flock comprised approximately 5,000 birds, with the affected group showing an estimated morbidity of 15% and a mortality rate of ~8% among 10-day-old ducklings, presenting primarily with lethargy and hepatic necrosis. The collected liver samples were individually homogenized (approximately 0.05 g per sample) for subsequent analysis. Following three cycles of freezing and thawing, the homogenates were centrifuged at 5,000 × *g* for 10 min to collect the supernatant for subsequent viral isolation and RNA extraction. Total RNA was extracted from each sample using the Ultrapure RNA Kit (CWBIO, CW0581M), and complementary DNA (cDNA) was synthesized using *EasyScript*® Reverse Transcriptase (M-MLV, RNaseH-) (TransGen, AE101) in accordance with the manufacturer’s instructions. The cDNA was amplified by PCR with specific primers (Table 1) targeting major waterfowl pathogens, including novel duck reovirus (NDRV), duck hepatitis virus (DHV), muscovy duck reovirus (MDRV), duck tembusu virus (DTMUV), duck plague virus (DPV), fowl adenovirus (FAdV), avian influenza virus (AIV), and duck circovirus (DuCV). Samples testing positive for NDRV were inoculated into 9-day-old specific pathogen-free (SPF) duck and chicken embryonated eggs via the chorioallantoic membrane (CAM) route. Inoculated embryos were incubated at 37 °C for 5 days and monitored twice daily for viability. Allantoic membranes and fluids were aseptically harvested from embryos that died within 24 h post-inoculation (h.p.i.) and subsequently passaged in duck embryos. Consistent positivity across samples confirmed the successful isolation of NDRV, and subsequent sequencing analysis indicated that all five isolates represented the same viral strain, designated SD416.

**Table 1:** Primers for detecting viral RNAs from clinically infected duck samples and amplification of the isolate SD416.

Primers	Sequence (5′ → 3′)	Product size (bp)
NDRV-JC-F	ACGCCTGACTACGATTACG	515
NDRV-JC-R	TCCGCTGCCCAAATGAATG	
NDRV-σC-F	ATGGATCGCAACGAGGTGAT	966
NDRV-σC-R	CTAGCCCGTGGCGACGGT	
MDRV-F	TCGTGCCTGTTTGTGGTG	349
MDRV-R	CCCGGACAGTCTTAATGTGA	
MDPV-F	ATGGCGGGTCTCAATCCA	390
MDPV-R	TTAGGTGTCGATGCCGGT	
DPV-F	GAAGCATATCGTTCGGAGGAG	376
DPV-R	TATCGCCTGCCAACTTATATCG	
DTMUV-F	CTCCGTCTTGGCATTAT	418
DTMUV-R	TCCTCAACCGCTTCC	
AIV-F	GATTGTAGTGTAGCTGGATGGCT	531
AIV-R	CCAGAAGAACTCCATTCTTCCAC	
FAdV-F	TAGTGATGCCGGGACATC	884
FAdV-R	TAGTGATGCCGGGACATC	
DuCV-F	GGGTGCCAATGGTCAG	333
DuCV-R	CACTGGGAAGCCCTCTA	
DHV-F	AAGAAGGAGAAAATCAAGGAAGG	467
DHV-R	TTGATGTCATAGCCCAACACAGC	
SD416-L1-F	GCTTTTTCTCCGAACGCCGAAAT	3,959
SD416-L1-R	GATGAGTAACCTCCAACGAGAGTCG	
SD416-L2-F	GCTTTTTCCTCACCATGCATGT	3,830
SD416-L2-R	GATGAATAATTCCTCGAGCCATGC	
SD416-L3-F	GCTTTTTCACCCATGGCTCAG	3,907
SD416-L3-R	GATGAGTAACACCCTTCTACTGGAGG	
SD416-M1-F	GCTTTTTCTCGACATGGCCTATCT	2,284
SD416-M1-R	GATGAGTATCTCAAGACGACTAACCCA	
SD416-M2-F	GCTTTTTGAGTGCTAACCTTTCTCACAC	2,158
SD416-M2-R	GATGAGTAACGTGCTAACCCAGAGAG	
SD416-M3-F	GCTTTTTGAGTCCTAGCGTGGATCA	1996
SD416-M3-R	GATGAATAACCGAGTCCGCCG	
SD416-S1-F	GCTTTTTTCTTCTCTGCCCATGGC	1,568
SD416-S1-R	GATGAATAGCTCTTCTCATTGCGCGC	
SD416-S2-F	GCTTTTTCTTCCACGATGGCGC	1,324
SD416-S2-R	GATGAGTACATCCACGTGCTGCC	
SD416-S3-F	GCTTTTTTGAGTCCTTAGCGTGC	1,202
SD416-S3-R	GATGAATAAGTGAGTCCCGCTAACC	
SD416-S4-F	GCTTTTTGAGTCCTTGTTCAGCCAT	1,191
SD416-S4-R	GATGAGTAAGAGTCCAAGTCGTGGC	

### Virus purification and transmission electron microscopy

The cell lysates were purified via ultracentrifugation at 8,000 × *g* for 30 min at 4 °C. The resulting supernatant was transferred to high-speed centrifuge tubes and subjected to further centrifugation at 25,000 × *g* for 2 h at 4 °C. The pellet was collected and resuspended in phosphate-buffered saline (PBS, pH 7.4). Following negative staining with 2% phosphotungstic acid, the samples were examined using a JEM-1400Plus transmission electron microscope (JEOL, Japan) for visualization of virions.

### Growth characteristics and virus titer determination

The third passage of duck embryo allantoic fluid was diluted 1:20 and used to inoculate monolayers of Vero and DF-1 (a chicken fibroblast cell line). Infected cells were maintained at 37 °C under 5% CO₂ and monitored daily for changes in cell morphology and the development of cytopathic effects (CPE) over a 5-day period. When CPE progression in Vero cells exceeded 80%, the cultures were subjected to three freeze–thaw cycles. The supernatant was then collected by centrifugation at 1,600 × *g* for 5 min. Serial dilutions of the viral suspension were prepared and used to infect Vero cells cultured in 96-well plates. The 50% tissue culture infectious dose (TCID₅₀) was determined using the Reed–Muench method (Niu et al., 2018b).

### Animal challenge experiment

The pathogenicity of SD416 was evaluated in ducklings of different ages. A total of 31-day-old SPF ducklings were randomly divided into four groups (groups I, II, III, *n =* 5 per group; group IV, *n =* 15) and housed in separate isolation units with ad libitum access to feed and water. Ducklings in group I were inoculated intramuscularly with 0.1 mL of SD416 viral suspension (1 × 10^6^ TCID₅₀/0.1 mL) at 1 day of age, while groups II and III received the same dose at 7 and 14 days old, respectively (Wang et al., 2019; Zhu et al., 2023). Group IV served as the age-matched negative control and was administered 0.1 mL of phosphate-buffered saline (PBS) via the same route. The ducklings were monitored daily for clinical signs until 7 days post-infection (dpi). Body weight was recorded, and throat and cloacal swabs were collected at 1, 3, 5, and 7 dpi. Ducklings that died during the trial were necropsied immediately, and samples of spleen and liver were collected for viral RNA detection by real-time quantitative PCR (RT-qPCR). At 7 dpi, all surviving ducklings were euthanized by cervical dislocation following deep surgical anesthesia induced with an intramuscular injection of Telazol (tiletamine-zolazepam) at 30 mg/kg body weight. The absence of pedal and corneal reflexes was confirmed prior to the procedure to ensure an adequate depth of anesthesia. Necropsy was then performed on all subjects. Tissue samples, including heart, spleen, liver, lung, kidney, thymus, pancreas, and bursa of Fabricius, were collected for viral load detection using RT-qPCR. In parallel, samples of liver and spleen were immersion-fixed in 10% neutral buffered formalin for histopathological processing. Following fixation, tissues were embedded in paraffin, sectioned, and stained with hematoxylin and eosin (HE) for detailed microscopic evaluation.

### Complete genome segment amplification and sequencing

The complete genome of strain SD416 was amplified using primers designed based on conserved regions of duck reovirus sequences available in GenBank (Table 2). PCR was performed under the following conditions: initial denaturation at 95 °C for 3 min; 35 cycles of denaturation at 95 °C for 10 s, annealing at 56 °C for 30 s, and extension at 72 °C for 1 min; followed by a final extension at 72 °C for 10 min. The amplified products were purified using a gel extraction kit (CWBIO, CW2302) and subsequently cloned into a blunt-ended vector using the *pEASY*®-Blunt Simple Cloning Kit (TransGen, CB111) according to the manufacturer’s protocol. For each genomic segment, three independent positive clones were selected and submitted to Sangon Biotech (Shanghai) Co., Ltd. for Sanger sequencing.

**Table 2:** Genetic characteristics of duck reovirus strain SD416.

Genome segment	Size (nt)	5’UTR	ORF	3’UTR	Encoded protein and size (aa)	Strain with highest nucleotide similarity (%)	Strain with highest amino acid similarity (%)
L1	3,959	1–21	22–3,903	3,904–3,959	λA (1293)	HD22 (99.92)	SY (99.85)
L2	3,830	1–14	15–3,794	3,795–3,830	λB (1259)	XT18 (99.37)	QAU-001 (99.84)
L3	3,907	1–12	13–3,870	3,871–3,907	λC (1285)	YN22 (98.86)	CD200801 (99.61)
M1	2,284	1–13	14–2,212	2,213–2,284	μA (732)	QAU-001 (99.32)	HD22 (99.73)
M2	2,158	1–29	30–2057	2058–2,158	μB (675)	K231 (99.36)	LY20 (99.85)
M3	1996	1–24	25–1932	1933–1996	μNS (635)	SDYC (98.9)	SDYC (99.69)
S1	1,568	1–19	20–313	1,537–1,568	P10 (97)	LY20 (99.66)	XT18 (98.97)
			273–761		P18 (162)	LY20 (99.59)	SDYC (99.38)
			571–1,536		σC (321)	LY20 (99.59)	LY20 (99.69)
S2	1,324	1–15	16–1,266	1,267–1,324	σA (416)	LY20 (99.36)	J18 (99.76)
S3	1,202	1–30	31–1,134	1,135–1,202	σB (367)	YL (99.37)	XT18 (99.18)
S4	1,192	1–23	24–1,127	1,128–1,191	σNS (367)	LY20 (99.55)	LY20 (99.73)

### Analysis of nucleotide and deduced amino sequences

The resulting 10 nucleotide sequences were assembled and aligned with other reovirus genomes via the MUSCLE algorithm implemented in MEGA-X software. A phylogenetic tree was reconstructed using the Maximum Likelihood (ML) method within the same platform, with branch support assessed through 1,000 bootstrap replicates. The viral open-reading frames (ORFs) prediction, sequence alignment and amino acids (aa) translation were conducted using the EditSeq and MegAlign modules of the DNASTAR Lasergene 12 Core Suite. The visual analysis of the entire genome alignment of the isolate strain and 11 reference strains was carried out on the mVISTA online platform.^1^

### Recombination detection

To detect potential recombination events, initial screening was performed using the Phi test implemented in SplitsTree software (Huson, 1998). Sequence alignments yielding a statistically significant signal (*p <* 0.05) were subsequently analyzed in depth using the RDP4 software suite (Martin et al., 2015). Within RDP4, we employed multiple independent detection algorithms (including RDP, GENECONV, BootScan, MaxChi, Chimaera, SiScan, and 3SEQ) (Arenas and Posada, 2010; Boni et al., 2007; Gibbs et al., 2000; Martin and Rybicki, 2000; Padidam et al., 1999; Salminen et al., 1995; Wiuf et al., 2001). A recombination event was considered robust and designated for further characterization only if it was consistently supported by at least three different methods with a default *p*-value threshold of < 0.05. For each putative event identified, the software estimated recombination breakpoints. Recombinant sequences and the identified recombination regions were then removed from the dataset to generate a final refined alignment for subsequent phylogenetic and evolutionary analyses.

### Statistical analysis

All statistical analyses in this study were conducted using one-way analysis of variance (ANOVA) or Student’s *t*-test in GraphPad Prism 8.0 (GraphPad Software, San Diego, CA, USA). Differences between groups were considered statistically significant at *p <* 0.05.

## Results

### Virus isolation and identification

**Figure 1:**
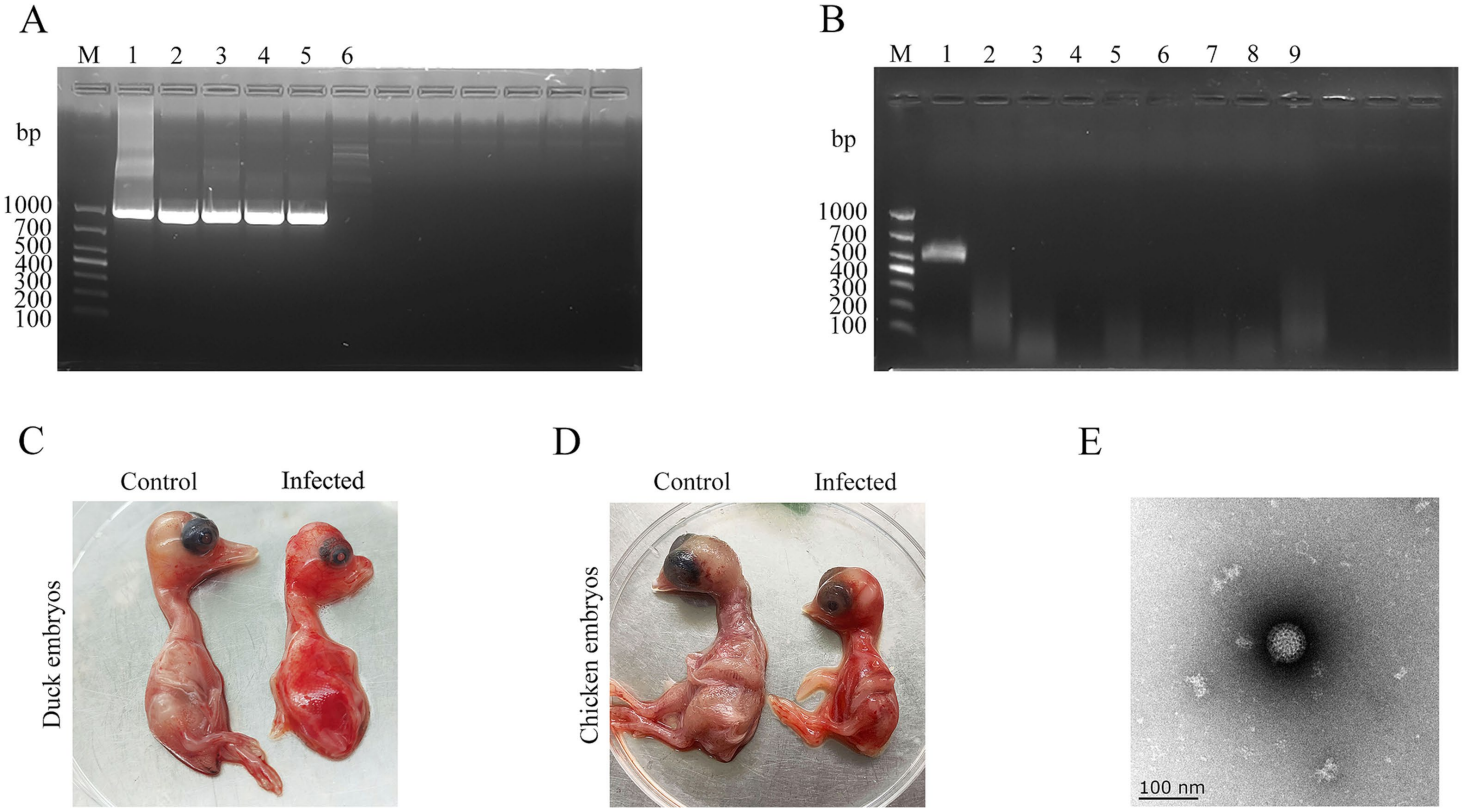
Isolation and identification of SD416. **(A)** PCR identification. M, DL1000 DNA marker; 1–5, SD416; 6, negative control. **(B)** Viral purity assessment. M, DL2000 DNA Marker; 1, SD416; 2–8, tests for DHV, MDRV, DTMUV, DPV, FAdV, AIV, and DuCV; 9, negative control; **(C)** Lesions in duck embryos following inoculation with SD416. **(D)** Lesions in chicken embryos inoculated with SD416. **(E)** Transmission electron micrograph of purified SD416 virions (100,000 × magnification). The virions exhibit typical non-enveloped, icosahedral symmetry. The average diameter was determined to be approximately 70 nm by measuring 50 randomly selected, intact particles using ImageJ software (version 1.53).

**Figure 2:**
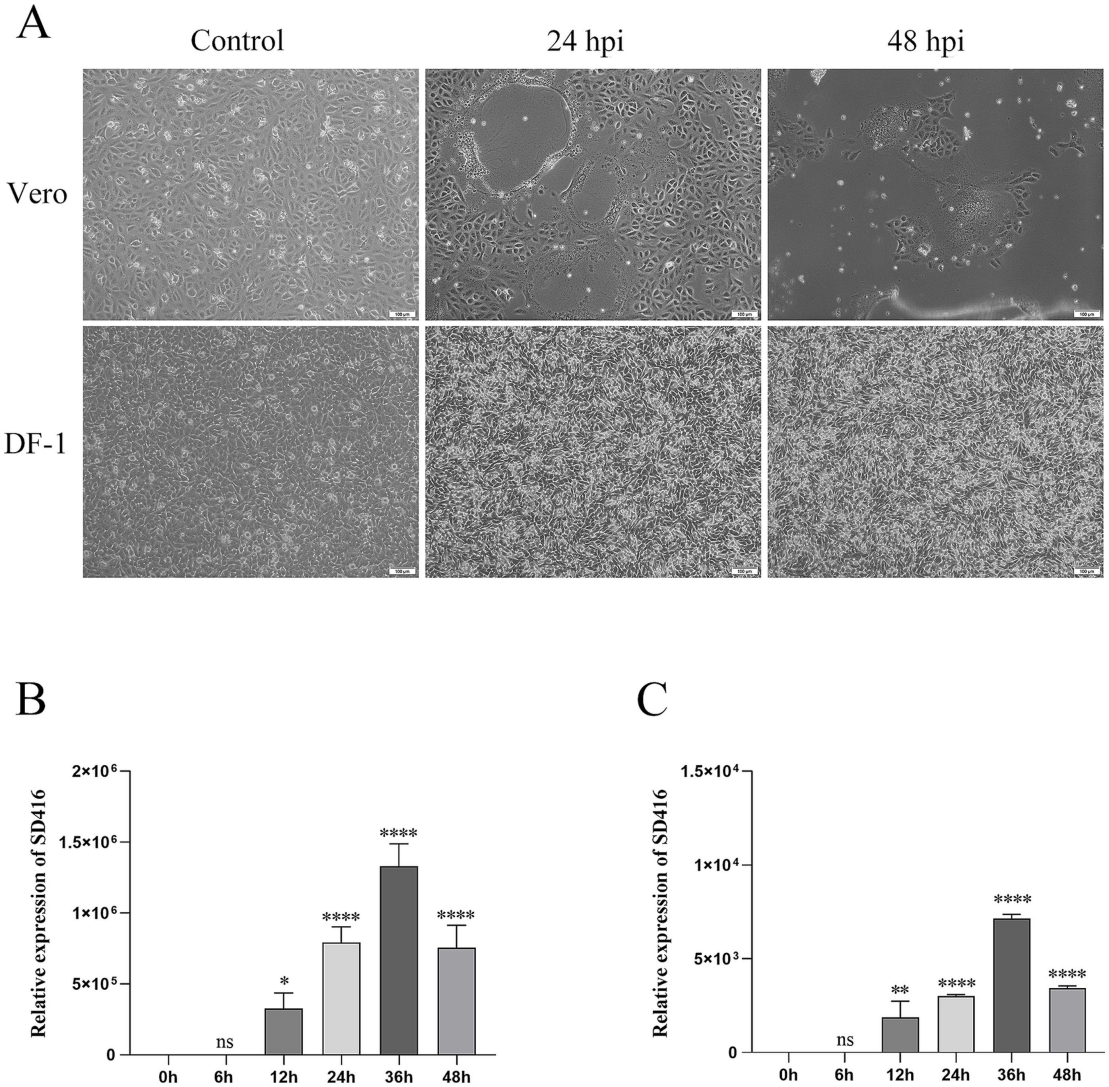
Replication kinetics of SD416 *in vitro*. **(A)** Cytopathic effects (CPE) in Vero and DF-1 cells at 24 and 48 h.p.i. **(B,C)** Viral replication levels in Vero and DF-1 cells infected with SD416, as determined by RT-qPCR. Data are presented as mean ± standard deviation (SD). Significance was determined by one-way ANOVA (**p < 0.01, ***p < 0.001, ****p < 0.0001; ns, not significant) comparing each time point to the mock-infected control.

As shown in Figure 1, all five liver tissue samples tested positive for novel duck reovirus (NDRV) by RT-PCR. Sequencing of the amplified products confirmed that all five samples contained an identical viral strain, designated SD416. Furthermore, PCR screening with specific primers (Table 1) confirmed the absence of other common duck pathogens, including duck hepatitis virus (DHV), Muscovy duck reovirus (MDRV), duck Tembusu virus (DTMUV), duck plague virus (DPV), fowl adenovirus (FAdV), avian influenza virus (AIV), and duck circovirus (DuCV). All inoculated specific pathogen-free (SPF) duck and chicken embryos died within 72–96 h during the second passage (Figures 1C,D), exhibiting severe subcutaneous hemorrhage. Virus particles were purified via sucrose density gradient centrifugation and negatively stained with phosphotungstic acid for transmission electron microscopy. The virions exhibited spherical, non-enveloped icosahedral symmetry, with a diameter of approximately 70 nm, as determined by measuring 50 randomly selected, intact particles using ImageJ software (version 1.53) (Figure 1E) (Spandidos and Graham, 1976). To assess cellular tropism and syncytium formation, Vero and DF-1 cell lines were infected with SD416. CPEs—characterized by cell rounding, increased refractility, and detachment—were observed in Vero cells within 24–48 h.p.i. In contrast, CPEs in DF-1 cells were not apparent until 120 h.p.i. (Figure 2A). RT-qPCR analysis of viral replication kinetics revealed that SD416 reached peak titers at 36 h.p.i. in both Vero and DF-1 cells (Figures 2B,C). These morphological and cytopathic features are consistent with those of reoviruses.

### Clinical symptoms and necropsy changes in NDRV-infected ducks

**Figure 3:**
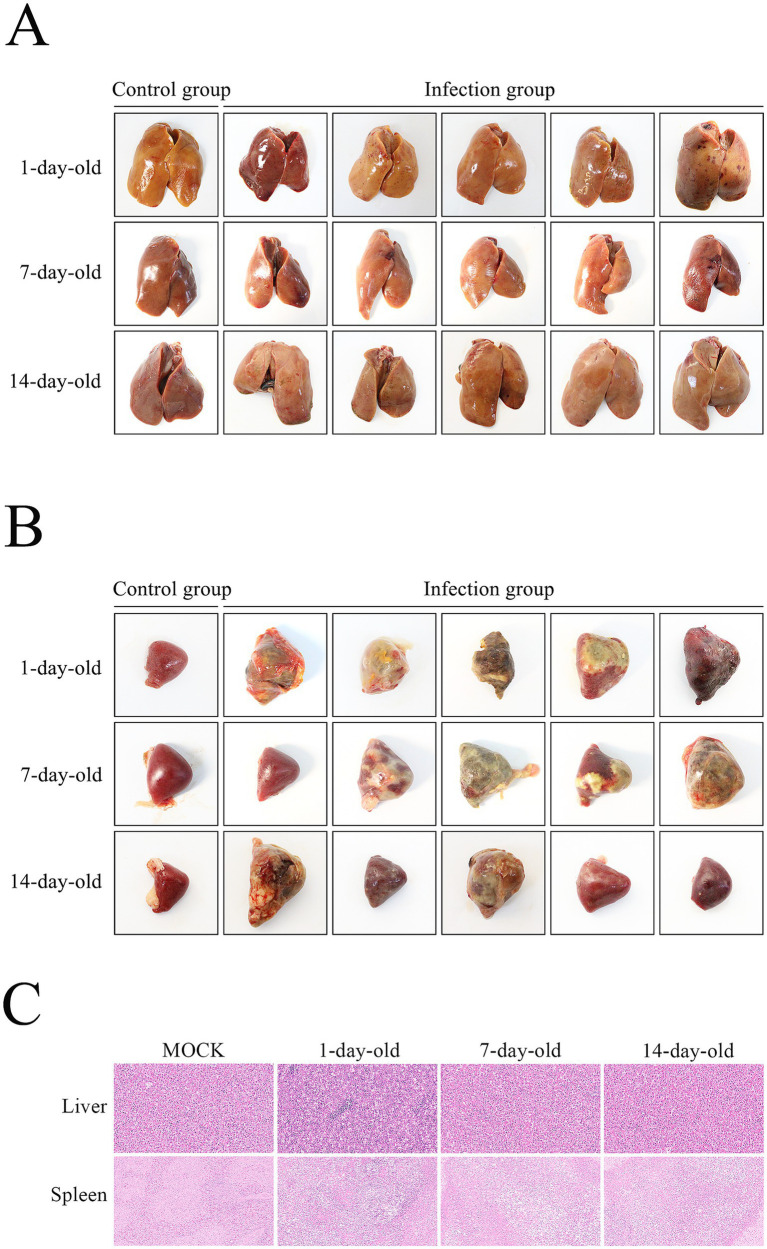
Pathological lesions in ducklings infected with SD416. **(A)** Liver lesions in 1-, 7-, and 14-day-old infected ducklings. **(B)** Splenic lesions in ducklings of different ages. **(C)** Histopathological analysis of liver and spleen tissues (HE staining, 200×). Liver sections show hemorrhage and hepatocellular swelling (absent in 7- and 14-day-old groups). Splenic sections exhibit architectural disruption and lymphoid depletion.

To evaluate the age-dependent pathogenicity of SD416, ducklings of different ages were intramuscularly inoculated and monitored for clinical signs and gross lesions. All challenged ducklings exhibited clinical symptoms, including lethargy, anorexia, and depression. Mortality was exclusively observed in Group I (1-day-old), which experienced two deaths concomitant with more severe clinical manifestations. No abnormalities were detected in the control group. Gross examination revealed hemorrhagic foci on the liver surface in 1-day-old infected ducks, but not in older groups (Figure 3A). All 1-day-old infected ducks exhibited splenic lesions including swelling, hemorrhage, yellow-white necrotic foci, and in severe cases, complete necrosis, with a lesion incidence of 100% (Figure 3B). Although 7- and 14-day-old infected ducks also showed splenic swelling and necrosis, the incidence and severity of lesions were reduced (80 and 60%, respectively). No lesions were observed in control ducks. Histopathological analysis confirmed marked lesions in the liver and spleen (Figure 3C). Hepatocellular congestion and swelling were prominent in 1-day-old ducks, but absent in older groups. The splenic architecture showed partial disruption and lymphoid depletion, with lesion severity decreasing with age. These results indicate that the severity of hepatic and splenic lesions induced by SD416 is negatively correlated with host age. Ducklings at 1 and 7 days old exhibited higher susceptibility, as evidenced by more frequent and severe splenic pathology compared to 14-day-old ducks.

**Figure 4:**
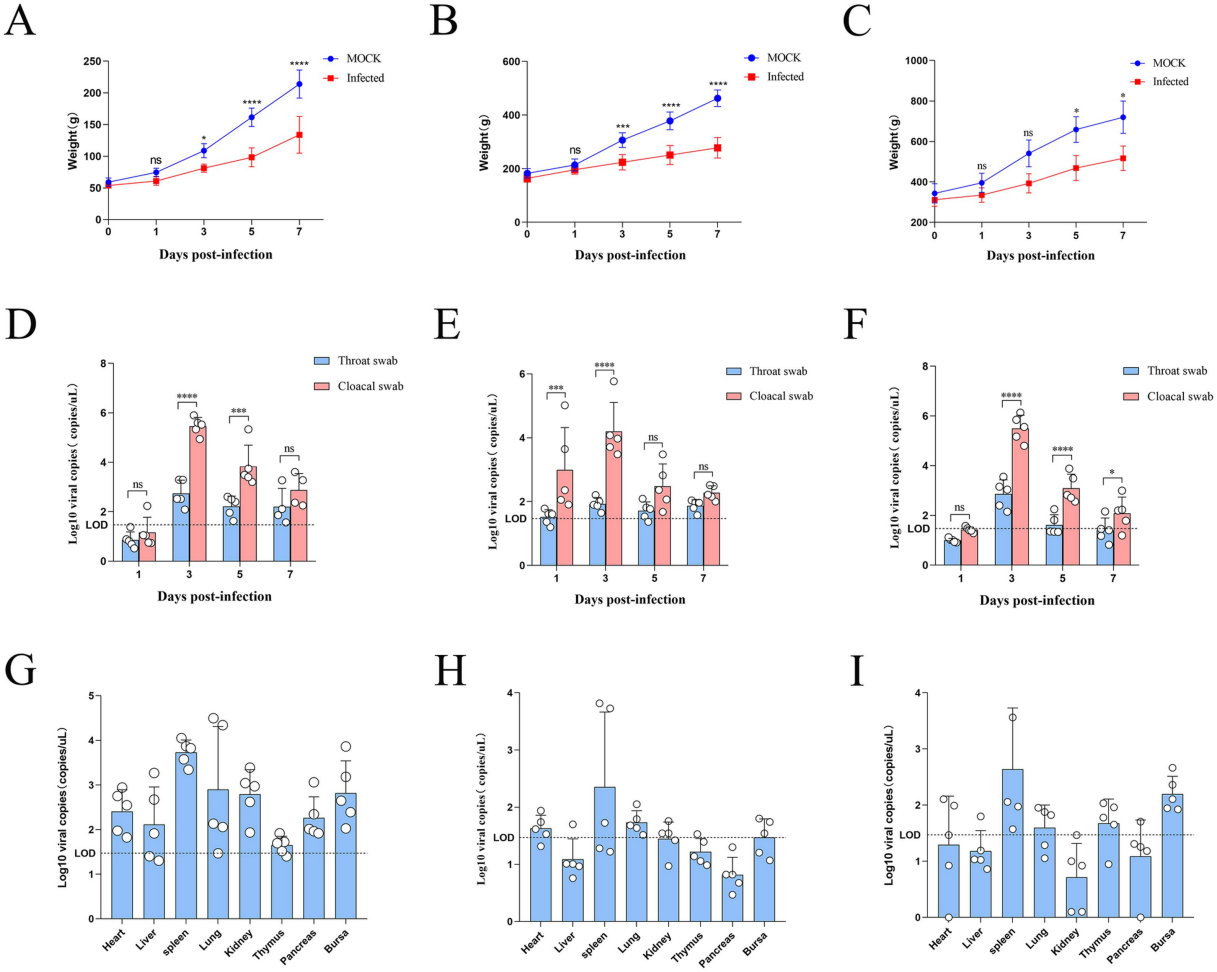
Pathogenicity of SD416 in ducklings of different ages. **(A–C)** Body weight changes in infected and control ducklings at 1, 7, and 14 days of age. Data at each time point were compared using an unpaired Student’s *t*-test versus the age-matched control group (*p < 0.05, **p < 0.01, ***p < 0.001, ****p < 0.0001). **(D–F)** Viral shedding levels in throat and cloacal swabs from ducklings infected at 1 **(D)**, 7 **(E)**, and 14 **(F)** days of age. Within each age group, statistical comparisons between throat and cloacal swabs at each time point were performed using paired Student’s t-tests with Holm-Šídák correction for multiple comparisons (*p < 0.05, **p < 0.01, ***p < 0.001, ****p < 0.0001). **(G–I)** Viral loads in various organs of infected ducklings at 7 d.p.i.

The body weight gain was significantly reduced in 1- and 7-day-old infected ducks at 5, 7 dpi compared to controls, while 14-day-old ducks showed only mild reduction (Figures 4A–C). Viral loads in cloacal and throat swabs were quantified by RT-qPCR. Viral shedding peaked at 3 dpi in all infected groups and was significantly higher in cloacal than in throat swabs (*p <* 0.01, unpaired *t*-test), suggesting fecal–oral transmission as the primary route (Figures 4D–F). Tissue tropism was also age-dependent. In 1-day-old ducks, the virus was detected in the heart, liver, spleen, lung, kidney, thymus, pancreas, and bursa of Fabricius, with the highest load in the spleen (Figure 4G). Viral loads in 7- and 14-day-old ducks were significantly lower and largely restricted to the spleen (Figures 4H,I), indicating that SD416 replicates most efficiently in splenic tissue and that viral replication competence decreases with host age. Together, these findings demonstrate that age-related resistance to SD416 is associated with reduced viral replication and limited tissue dissemination, underscoring the critical role of host age in susceptibility to NDRV infection.

### Global distribution of reovirus

**Figure 5:**
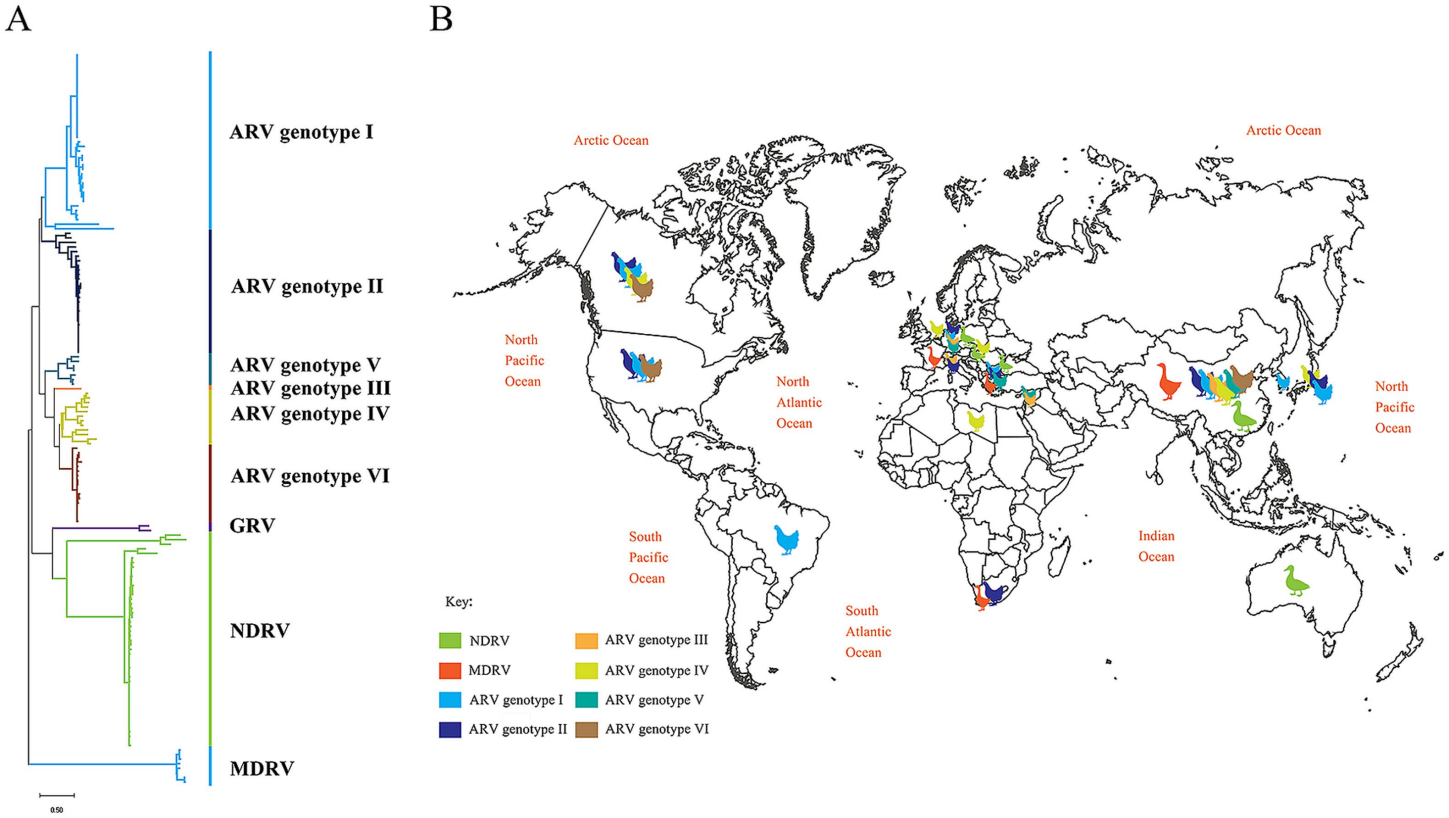
Phylogenetic and global distribution analysis of avian reoviruses. **(A)** Maximum likelihood tree based on σC gene sequences, constructed using MEGA-X with 1,000 bootstrap replicates. The SD416 strain is indicated by a green triangle. **(B)** Global distribution of avian reovirus genotypes. Colors correspond to NDRV, MDRV, and ARV genotypes I–VI.

The σC gene is widely recognized as a genetic marker for the differentiation and classification of Orthoreovirus isolates. To elucidate the evolutionary relationships based on the σC gene, we obtained and analyzed 160 waterfowl reovirus (WRV) sequences. Phylogenetic analysis revealed that these sequences formed four distinct clusters: 103 isolates grouped within the avian reovirus (ARV) branch, 47 within the novel duck reovirus (NDRV) branch, 8 within the Muscovy duck reovirus (MDRV) branch, and 2 within the goose reovirus (GRV) branch. Furthermore, the 103 ARV σC sequences were subdivided into six subclades: subclade I (39 sequences), subclade II (27 sequences), subclade III (1 sequence), subclade IV (12 sequences), subclade V (7 sequences), and subclade VI (17 sequences) (Figure 5A). Strain SD416 clustered within the NDRV branch alongside most Chinese isolates and showed a close phylogenetic relationship with strain N-DRV/LY20.

WRVs are distributed globally. Avian reovirus was first identified in 1954 at the Connaught Medical Research Laboratory, University of Toronto, Canada (Fahey and Crawley, 1954). Since then, ARVs have been reported across the Americas (United States and Brazil), Europe (the Netherlands, Germany, Hungary, France, Spain, and Switzerland), Asia (China, South Korea, Japan, and Israel), Oceania (Australia), and Africa (South Africa and Egypt) (Figure 5B). In China, Wang Xikun first described the disease in 1985. In 1991, Chen Likuan and colleagues isolated avian reovirus from chickens exhibiting lameness and joint swelling, confirming its presence at the pathogen level. Subsequent cases were reported in multiple provinces. Muscovy duck reovirus (MDRV) was initially reported by Kaschula in Africa and has since been identified in Europe (France) and Asia (China). In China, the disease was observed in Muscovy ducklings in Zhejiang and Fujian provinces beginning in 1997 and was confirmed as MDRV by Wu Baocheng et al. in 2001. Subsequent cases in hybrid ducks and geese were documented by Huang Yu, Wang Yongkun, and others. Novel duck reovirus (NDRV) was first detected in 2005 in Fujian Province (Putian, Fuzhou, and Changle) and subsequently became endemic in major waterfowl-producing regions such as Guangdong and Zhejiang (Figure 5B). In 2007, Liu Hong et al. isolated a reovirus strain, DRV-GZ, from Beijing ducks showing symptoms including yellowish-green diarrhea, head swelling, leg weakness, ocular discharge, hepatic necrosis and hemorrhage, and esophageal and cloacal ulcers. This represented the first confirmed case of reovirus infection in Beijing ducks with pathogen isolation. In 2009, Chen Shaoying et al. isolated three reovirus strains from diseased Shelducks in Fujian province presenting irregular hepatic necrosis and hemorrhage. Thereafter, NDRV reports emerged successively across multiple regions (Chen et al., 2012).

### The complete genome of SD416

The complete genomic sequences of segments 1–10 of strain SD416 were amplified by RT-PCR using specifically designed primers, and the terminal sequences (5′ and 3′) of each segment were determined using rapid amplification of cDNA ends (RACE). The full genome of SD416 was determined to be 23,420 bp in total length (Table 2). The complete genome sequence has been deposited in GenBank. The sizes of the 10 segments are as follows: L1 (3,959 bp), L2 (3,830 bp), L3 (3,907 bp), M1 (2,284 bp), M2 (2,158 bp), M3 (1,996 bp), S1 (1,568 bp), S2 (1,324 bp), S3 (1,202 bp), and S4 (1,192 bp). Open reading frame (ORF) prediction revealed that all segments except S1 are monocistronic, encoding the proteins *λ*A, λB, λC, μA, μB, μNS, σA, σB, and σNS, respectively. The S1 segment contains three partially overlapping ORFs encoding P10, P18, and σC (Table 2).

Each protein sequence of SD416 was used as a query in BLASTp homology searches. The strain N-DRV/LY20 showed the highest amino acid identity with the corresponding proteins of SD416: λA (99.67%), λB (99.06%), λC (98.29%), μA (98.21%), μB (99.26%), μNS (98.50%), P10 (99.66%), P18 (99.59%), σC (99.59%), σA (99.40%), σB (98.17%), and σNS (99.58%) (Table 2).

### Phylogenetic analysis and sequence comparison

**Figure 6:**
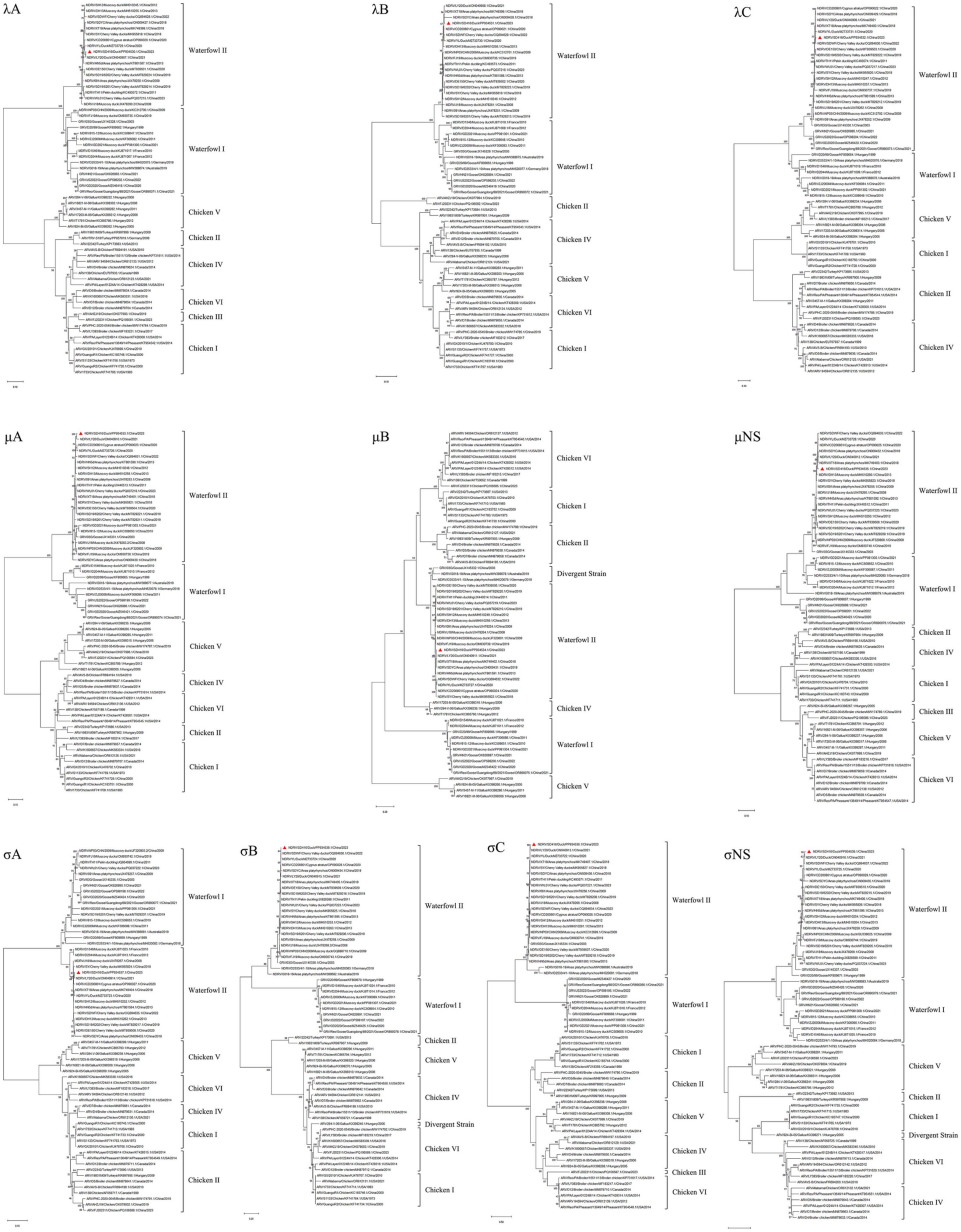
Phylogenetic analysis of SD416 based on complete ORFs of 10 genomic segments. Maximum likelihood trees were inferred using MEGA-X with 1,000 bootstrap replicates. The SD416 strain is marked with a red triangle.

To elucidate the evolutionary relationships between the novel duck virus identified in this study and other orthoreoviruses, phylogenetic trees were constructed based on the open reading frames (ORFs) of all 10 genomic segments using the maximum-likelihood (ML) method with 1,000 bootstrap replicates. Each segment displayed distinct clustering patterns, forming two major clades corresponding to different genogroups: chicken-associated and waterfowl-associated. In accordance with established avian reovirus (ARV) genotyping criteria, the waterfowl genogroup could be further subdivided into two genotypes: waterfowl genotype I (MDRV and GRV) and waterfowl genotype II (represented by NDRV). Phylogenetic analysis consistently placed the SD416 isolate within the NDRV lineage (genotype II), indicating that it represents a newly emerged Chinese orthoreovirus strain distinct from previously characterized avian reoviruses (Figure 6).

Genetic recombination among viral segments contributes to genomic diversity, and viral classification often relies on hypervariable gene regions. In waterfowl reoviruses, longer genomic segments generally exhibit lower genetic variability compared to medium and small segments. Based on λ-class gene phylogenies, the SD416 strain showed close evolutionary relationships with LY20, YL, and CD200801 strains in the λA and λC genes. In contrast, significant divergence was observed in the λB gene among these strains—including LY20, YL, CD200801, and SDYC—despite their overall clustering within the same major branch, suggesting ongoing viral divergence (Figure 6, λA–λC). Analysis of the M-class segments indicated that SD416 clusters with LY20, XT18, and SDYC strains in both μB and μNS genes. However, the μA gene displayed a distinct phylogenetic pattern: although SD416 and LY20 formed a monophyletic clade, they diverged significantly from XT18 and SDYC within this segment (Figure 6, μA–μNS). Among *σ*-class genes, the greatest phylogenetic divergence was observed. The σC gene, which encodes the major neutralization antigen and cell attachment protein of waterfowl reoviruses, exhibited the most pronounced evolutionary separation among all 10 genomic segments, indicating that SD416 has undergone a unique evolutionary trajectory. In contrast, phylogenies of σA and σNS genes showed SD416 forming a clade with LY20, consistent with topologies observed for λA, μA, and μB segments. This concordance across multiple genomic regions suggests a shared evolutionary origin and functional conservation among these strains (Figure 6, σA–σNS).

Collectively, the phylogenetic incongruence observed across the 10 genomic segments (Figure 6) indicates that strain SD416 may have undergone complex evolutionary events. The distinct clustering patterns, particularly the independent evolutionary trajectories of the λB, μA, and σC segments, provide strong genomic evidence suggestive of potential reassortment or segment-specific recombination with other circulating avian orthoreoviruses, rather than a simple linear descent from a single predecessor.

### Visualization of whole-genome alignment and analysis of amino acid sequences

**Figure 7:**
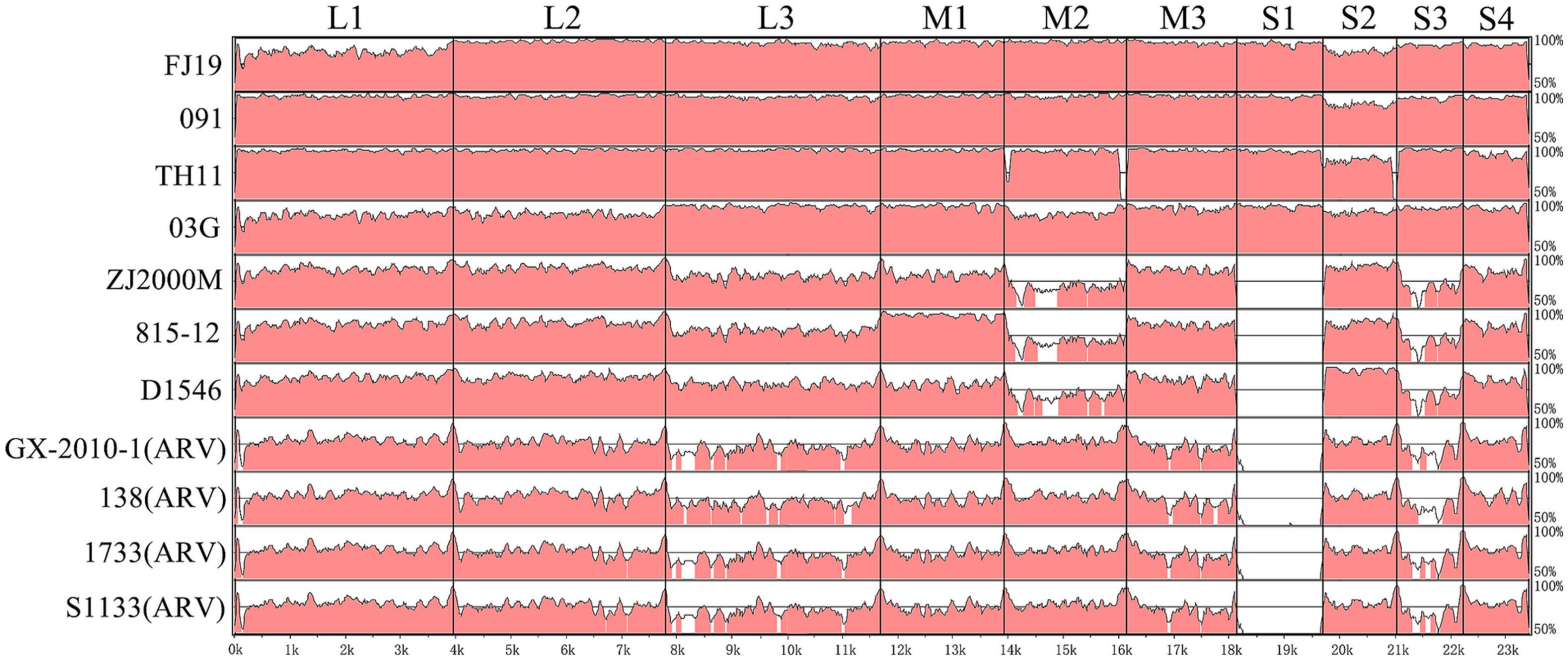
Visualization of the whole genome of the SD416. The alignment result of SD416 in comparisons with the FJ19, 091, TH11, 03G, ZJ2000M, 815–12, D1546, GX-2010-1, 138, 1733, and S1133 strains are illustrated. Areas in pink represent ≥95% similarity, and those in white represent <95% similarity.

Visualization of the genome revealed a series of divergent regions between the genome of the SD416 strain and other 11 orthoreovirus strains (Figure 7). The SD416 strain displayed low sequence similarity with the GX-2010-1, 138, 1733 and S1133 strains, indicating a considerable genetic distance from these avian orthoreoviruses. The SD416 strain shared high genetic relatedness to duck-origin strains FJ19, 091, TH11, and 03G, but with lower sequence similarity occurring in the L3, M2, and S3 segments compared to Muscovy duck strains (ZJ2000M, 815–12, D1546). This pronounced divergence was particularly critical in the S1 gene, which shared <50% sequence identity with classic MDRV and avian orthoreoviruses, thereby excluding their involvement in a recombination event leading to the emergence of SD416.

The visualization clearly demarcates SD416 from classical avian and Muscovy duck reoviruses (Figure 7). Most critically, the extensive divergence spanning the S1 segment, which encodes the major antigenic and cell-attachment protein σC, provides a visual and quantitative confirmation of the significant genetic shift represented by SD416. This large-scale divergence likely underpins the observed unique phenotypic characteristics and suggests substantial antigenic drift, which has direct implications for cross-protection and diagnostic assay design.

**Figure 8:**
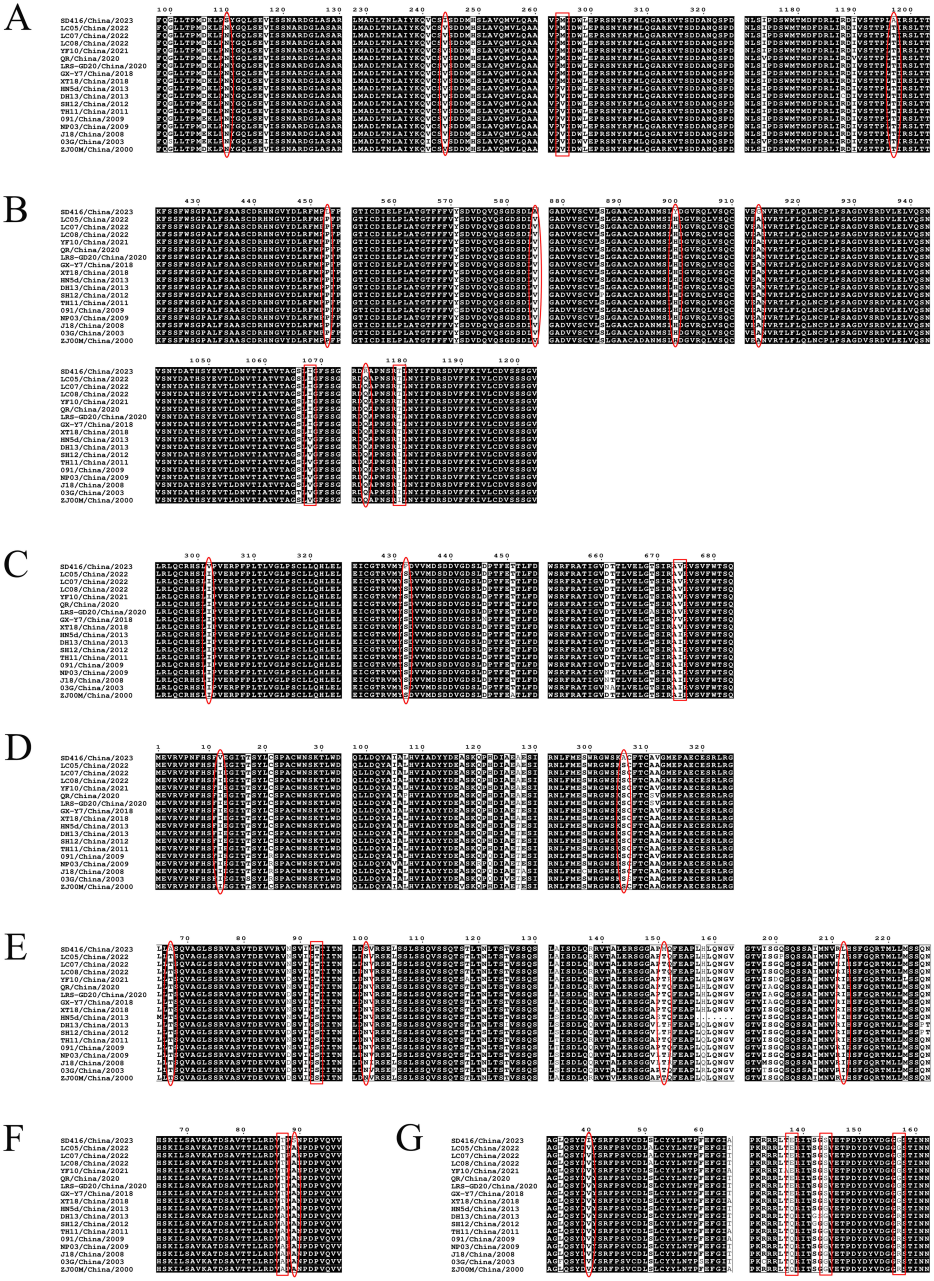
Multiple alignment of λB, λC, μA, σB, σC, P10, and P18 protein amino acid sequences from the SD416 strain. **(A)** Alignment of the λB protein aa sequences. **(B)** Alignment of the λC protein aa sequences. **(C)** Alignment of the μA protein aa sequences. **(D)** Alignment of the σB protein aa sequences. **(E)** Alignment of the σC protein aa sequences. **(F)** Alignment of the P10 protein aa sequences. **(G)** Alignment of the P18 protein aa sequences. (The red ellipse highlights regions where the isolate strain differs from all reference strains, and the red box indicates mutations specific to isolates obtained since 2018).

Comparative alignment of the λB, λC, μA, σB, σC, P10, and P18 proteins revealed that the SD416 strain diverged from 17 other Chinese NDRV strains at specific residues (Figures 8A–G). Key differences included three substitutions in λB (S110N, I244V, A1197T), five in λC (L453P, A585V, Y900H, G913A, R1173Q), two each in μA (V302I, P432S) and σB (V12I, A306S), four in σC (A67T, S100N, M151T, L212I), and one each in P10 (S89A) and P18 (I40V). Furthermore, isolates collected from 2018 and thereafter were characterized by a distinct set of mutations, including λB (295 M), λC (1068I, 1,179 T), μA (674 V), σC (93 T), P10 (87 T), and P18 (138E, 144S, 157G). Together, these findings collectively indicate that the accumulation of point mutations likely underlies the sequence divergence observed in the σC, P10, and P18 proteins between the SD416 strain and other NDRV strains, potentially contributing to their distinct phenotypic characteristics.

### Genetic recombination in reovirus

Genetic recombination serves as a critical mechanism driving viral evolution and genetic diversification. To examine potential intragenic recombination events among 64 waterfowl reovirus (WRV) isolates, we employed RDP4 and SimPlot 3.5.1 software to screen the 10 genomic segments of the NDRV SD416 strain alongside reference sequences. This multi-method analysis provided evidence suggestive of recombination in four segments: λB, μA, σA, and σNS.

**Figure 9:**
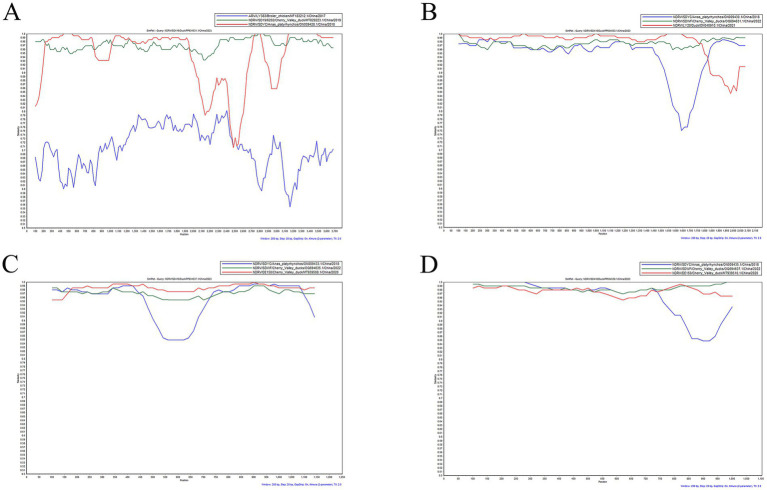
Recombination analysis of SD416 genomic segments. Putative recombination events in the λB, μA, σA, and σNS segments of strain SD416 were analyzed using RDP4 and visualized with SimPlot 3.5.1. Breakpoints supported by ≥3 algorithms (RDP, GENECONV, BootScan) were identified at: **(A)** λB (2030, 2634); **(B)** μA (1550, 1778); **(C)** σA (528, 653); **(D)** σNS (820, 958).

For each of these segments, the analysis identified specific breakpoint coordinates, which are provided in the legend of Figure 9. The patterns observed for the λB gene are consistent with a recombination event involving NDRV/SD19/6202/China/2019 (GenBank: MT829223.1) and ARV/LY383/China/2017 (GenBank: MF183212.1) as major and minor parents, respectively (Figure 9A). Similarly, the μA, σA, and σNS genes displayed phylogenetic and similarity plot signals indicative of potential recombination between various circulating NDRV and ARV strains (Figures 9B–D).

The analyses collectively indicate that the genome of SD416 may have been influenced by recombination between NDRV and ARV lineages.

## Discussion

The continual emergence of novel duck reovirus (NDRV) variants underscores the dynamic evolution of avian *orthoreoviruses* and poses significant challenges to global waterfowl health. This study characterizes a newly identified NDRV strain, SD416, which exhibits significant genetic divergence from previously reported isolates.

In this study, a reovirus strain, designated SD416, was isolated from liver tissues of diseased Cherry Valley ducks. The isolate induced mortality in both duck and chicken embryos and replicated efficiently in Vero and DF-1 cell lines. Transmission electron microscopy revealed non-enveloped viral particles with icosahedral symmetry and a double-layered capsid structure, consistent with typical avian *orthoreovirus* morphology. Whole-genome sequencing indicated that the SD416 genome comprises 23,420 bp, with segment lengths highly conserved relative to other known NDRV reference strains (Yang et al., 2023).

Our segment-by-segment phylogenetic analysis (Figure 6) revealed notable topological incongruences, which, beyond confirming the NDRV genotype, underscore the genomic plasticity of these viruses. The fact that genes encoding core polymerase components (λB), outer capsid proteins (μA, σC), and non-structural proteins (σNS) each tell a slightly different evolutionary story highlights that the evolution of waterfowl reoviruses is not monolithic. This mosaic genomic architecture is consistent with the hypothesis that novel variants like SD416 can emerge through the reassortment of genome segments from distinct parental strains co-circulating in waterfowl populations, thereby rapidly generating genetic and potentially phenotypic diversity.

The σC protein, which serves as the primary viral attachment protein for cell receptor binding, plays a crucial role in virus adsorption and syncytium formation. Notably, among the amino acid substitutions identified in the σC protein of SD416 (A67T, S100N, M151T, L212I), S100N and M151T are of particular interest. Structural alignment with known avian orthoreovirus σC proteins indicates that these residues are located within or adjacent to predicted receptor-binding domains and major antigenic epitopes. Specifically, these sites map to the C-terminal *β*-barrel head domain, which is responsible for receptor attachment, and overlap with regions identified as variant-specific immunodominant epitopes (Dawe et al., 2022; Guardado-Calvo et al., 2009). This positional information provides sequence-based support for the potential functional impact of these mutations. Based on this analysis, we hypothesize that the S100N and M151T substitutions may alter host cell tropism and facilitate escape from neutralizing antibodies elicited by existing vaccine strains, which could have implications for vaccine cross-protection. To directly test this hypothesis, future studies are warranted, including neutralization assays with strain-specific antisera, receptor-binding assays, and structural modeling/comparative analysis of the variant σC protein.

The whole-genome alignment visualization (Figure 7) further complements the phylogenetic and recombination analyses. It not only confirms the overall genetic distinctness of SD416 but also spatially maps the regions of highest variability. The pronounced divergence in the S1 segment, visually apparent in Figure 7, directly correlates with the unique set of mutations we identified in the σC, p10, and p18 proteins (Figure 8). This integrated view is consistent with the notion that selective pressures—possibly from host immunity—may be driving concentrated evolution in this region, which is critical for virus-host interaction. Therefore, Figure 7 is not merely a representation of distance but a map highlighting the genomic “hotspots” that likely govern the altered tropism and pathogenicity of emerging NDRV strains like SD416.

It is also noteworthy that significant genetic differences were observed in the M2 segments between SD416 and reference strains, suggesting that genomic studies of DRV should extend beyond the S-class genes. Interestingly, despite substantial nucleotide sequence divergence in the μB coding gene, the resulting amino acid changes between the isolate and reference strains were relatively conservative. Beyond point mutations, genetic recombination is a major driver of evolution for segmented dsRNA viruses, facilitating the emergence and spread of novel variants with altered virulence (Greenbaum et al., 2012).

Notably, our analyses provided evidence suggestive of recombination in the λB, μA, σA, and σNS genes of SD416. The mosaic genome of SD416 may thus reflect recombination between circulating strains. It is important to note that the observed phylogenetic incongruence could, in principle, also arise from evolutionary processes other than recent recombination, such as ancient lineage segregation or incomplete lineage sorting. Nevertheless, the detection of specific breakpoints supported by multiple algorithms makes genetic recombination the most parsimonious explanation; the genomic architecture of SD416 appears to have arisen through such events, and the inferred parental strains likely originated from distinct NDRV/ARV lineages. The λB and μA proteins, encoded by the L1 and M1 segments, are implicated in replicase activity, viral transcription, and genome binding (Su et al., 2007; Xu and Coombs, 2008). The σA protein, encoded by S2, antagonizes interferon-mediated antiviral innate immunity (Guardado-Calvo et al., 2008), while σNS, from S4, activates the PI3K/Akt signaling pathway—a mechanism linked to both viral replication and oncogenesis—by suppressing apoptosis and thereby enhancing viral persistence (Xie et al., 2016; Xie et al., 2020). Owing to its central role in receptor interaction and neutralization, the σC protein exhibited the highest genetic variability, consistent with its function-driven evolutionary plasticity. Although not observed in this study, prior reports indicate that reassortment involving the M2 segment can alter viral tropism (Wang et al., 2019; Wang W. et al., 2020). While further functional validation is needed, reassortment among circulating avian reoviruses may represent a significant mechanism underlying the emergence of novel poultry pathogens. Our phylogenetic and distribution analyses indicate that genetically similar NDRV strains have been reported in Europe (Germany and Hungary) and Oceania (Australia) (Figure 5B), suggesting a potential route for international dissemination via live poultry trade. However, direct sequence links require further surveillance and sharing of genomic data from these regions. Severe hepatic and splenic pathology, along with mortality, was observed in 1-day-old ducklings. In contrast, lesions in 7- and 14-day-old ducklings were confined to the spleen, with no mortality recorded. Histopathological examination of infected spleens showed pronounced cellular degeneration, hemorrhage, and necrosis. The highest viral loads were detected in splenic tissues, identifying the spleen as a primary site of viral replication. As a pivotal immune organ in birds, the spleen is essential for immunologic function (Wang et al., 2019). NDRV infection depletes splenic lymphocytes and induces immunosuppression, emphasizing the need for early intervention (Luo et al., 2021; Yun et al., 2018). Although 14-day-old ducklings showed relative resistance, high-dose challenge (10^6^ TCID₅₀/0.1 mL/duck) still induced splenic lesions in 80% of infected birds, suggesting that host age, viral dose, strain-specific virulence, and genetic background collectively influence disease outcomes (Wang H. et al., 2020).

The age-dependent resistance observed in this study, characterized by reduced splenic viral loads and limited systemic dissemination in older ducklings, likely reflects the maturation of the host immune system. The spleen, a primary lymphoid organ in birds, undergoes significant development in the first weeks post-hatching. Enhanced innate immune responses (e.g., interferon production, macrophage activity) and a more established adaptive immune repertoire in 7- and 14-day-old ducks may more effectively contain initial viral replication within the spleen, preventing the widespread viremia and severe multi-organ pathology seen in 1-day-old immunologically naive ducklings. Future studies profiling immune gene expression in the spleen during NDRV infection are warranted to test this hypothesis.

In summary, this study isolated and characterized a novel duck orthoreovirus, SD416, through whole-genome sequencing and phylogenetic analysis. Global distribution and age-dependent pathogenicity assessments enhance our understanding of NDRV molecular epidemiology and host adaptation. Collectively, our findings contribute to a foundation for developing targeted control strategies against NDRV. In the absence of effective vaccines or specific antiviral therapies, sustained surveillance and genetic characterization of circulating strains are essential for predicting and mitigating future outbreaks.

## Data Availability

The datasets presented in this study can be found in online repositories. The names of the repository/repositories and accession number(s) can be found at: https://www.ncbi.nlm.nih.gov/genbank/, PP934530–PP934539.

## Footnotes

1
http://genome.lbl.gov/vista/mvista/submit.shtml
